# Protocol for a randomized comparative effectiveness trial comparing a very low-carbohydrate diet to DASH diet for polycystic ovary syndrome: the SUPER (Supporting Understanding of PCOS Education and Research) trial

**DOI:** 10.1186/s13063-024-08583-y

**Published:** 2024-11-09

**Authors:** Sarah Greenwell, Aubree Jones, Yolanda R. Smith, Deanna Marriott, James E. Aikens, Vasantha Padmanabhan, Laura R. Saslow

**Affiliations:** 1 University of Michigan, Ann Arbor, MI USA; 2 Department of Health Behavior and Biological Sciences, School of Nursing, University of Michigan, Ann Arbor, MI USA; 3 Department of Obstetrics and Gynecology, University of Michigan, Ann Arbor, MI USA; 4 Department of Family Medicine, University of Michigan Medical School, Ann Arbor, MI USA; 5 Department of Pediatrics, University of Michigan, Medical Professional Building, Ann Arbor, MI USA

**Keywords:** Polycystic ovary syndrome, Glycemic control, Lifestyle intervention, Very low-carbohydrate diet, Ketogenic diet, DASH diet, Randomized comparative effectiveness trial

## Abstract

**Background:**

Polycystic ovary syndrome (PCOS), the most common endocrine disorder for women of reproductive age, is associated with increased risk for insulin resistance and type 2 diabetes. Current PCOS treatments insufficiently address the spectrum and severity of the disorder, and there is little evidence-based guidance available for lifestyle management of PCOS, especially through nutritional approaches. Some evidence shows that a very low-carbohydrate diet can improve glucose control compared to low-fat or moderate-carbohydrate diets, leading to improved glucose control and insulin levels that may help to treat symptoms of PCOS. This research investigates whether a very low-carbohydrate diet is more effective in improving glucose control and decreasing symptoms of PCOS in comparison to a DASH diet.

**Methods:**

The SUPER study aims to address the gap in knowledge about nutritional advice for people with PCOS through a randomized, comparative effectiveness trial comparing two approaches to glucose control: the dietary approaches to stopping hypertension (DASH) diet, and a very low-carbohydrate (VLC) diet. We will randomize 184 women with PCOS with body mass indexes (BMIs) between 25 and 50 kg/m^2^ to a VLC or DASH diet. All participants will follow a 24-session, 12-month, online diet, and lifestyle intervention that teaches their assigned diet. Participants will receive nutritional education, support from diet coaches, and education about behavioral strategies to improve dietary adherence. The primary outcome measure is HbA1c, and secondary outcomes include glucose variance, lipid and hormone levels (including total and free testosterone), PCOS symptoms, inflammation (measured by high-sensitivity C-reactive protein), body composition and weight, psychological well-being, and intervention feasibility and acceptability.

**Discussion:**

The SUPER study is a randomized comparative effectiveness trial that compares two promising approaches to glucose control in people with PCOS. The study also aims to assess the effects of each diet on PCOS symptoms. The research addresses an important gap in knowledge regarding nutritional advice for people with PCOS.

**Trial registration:**

ClinicalTrials.gov NCT05452642. Registered 6 July 2022.

## Administrative information

**Table:** 

**Title {1}**	The Supporting Understanding of PCOS Education and Research (SUPER) Study
Trial registration {2a and 2b}	ClinicalTrials.gov NCT05452642
Protocol version {3}	Jul 12, 2024 Version 1.26
Funding {4}	This trial is funded by NIH grant 5R01DK128205-02
Author details {5a}	University of Michigan, Ann Arbor, MI, USA
Name and contact information for the trial sponsor {5b}	The National Institute of Diabetes and Digestive and Kidney Diseases (NIDDK), National Institutes of Health (NIH; healthinfo@niddk.nih.gov; USA; 1–800-860–8747)
Role of sponsor {5c}	This is an investigator-initiated trial; the NIH was not involved in planning the trial design and will not be involved in the collection, management, analysis, and interpretation of data; writing of reports; or the decision to submit the report for publication

## Introduction

### Background and rationale {6a}

Polycystic ovary syndrome (PCOS) is the most common endocrine disorder for women of reproductive age, yet treatments for the condition are few and do not sufficiently address the spectrum and severity of symptoms [1]. The condition is associated with endocrine dysregulation and a higher risk of health complications in women with PCOS. Comorbid conditions and health complications associated with PCOS include type 2 diabetes, hyperandrogenism (leading to oligomenorrhea-anovulation, hirsutism, and acne), reproductive disorders (chronic anovulation, infertility), cancers, and cardiovascular disease, in addition to a higher likelihood of having beta-cell dysfunction [2–5].

PCOS has a yearly economic burden of 4.3 billion dollars in the USA, 40% of which is due to type 2 diabetes care [6]. In fact, women with PCOS have a 3–7 times higher risk for type 2 diabetes [3], and women with PCOS whose body weight is in the obesity range have an even higher risk [7–9]. Obesity and PCOS tend to co-occur; about 30–60% of women with PCOS are in the obesity range [10] and over 10% of obese women have PCOS [11]. Compared to other women with PCOS, women with PCOS who have a body mass index (BMI) in the obesity range have poorer reproductive and metabolic outcomes, including higher androgens, fasting glucose, fasting insulin, insulin resistance, and lipids [7, 8].

Women with PCOS may address their heightened risk of developing type 2 diabetes through methods that improve their glycemic control. Experts recommend that treatment for women with PCOS should include diet and lifestyle interventions to moderate glycemic control [12–17]. Experts disagree about the optimal nutritional advice for women with PCOS [18]. Some recommend no particular diet [19], others recommend the high-carbohydrate Dietary Approaches to Stop Hypertension (DASH) diet [17], and still others encourage a lower carbohydrate diet [20].

A systematic review of previous dietary trials in women with PCOS found a slight benefit of lower carbohydrate diets for glucose control, insulin resistance, and PCOS symptoms [20]. One potential explanation for this effect is that low-carbohydrate diets are not as insulinogenic as a standard diet, which may contribute to decreased hyperandrogenism and associated symptoms in people with PCOS. Carbohydrate intake raises glucose, which in turn increases insulin secretion. High insulin stimulates more ovarian androgen production — leading to worsened symptoms of hyperandrogenism — and inhibits the release of fatty acids from cells [21, 22]. Based on this potential mechanism, decreasing carbohydrate intake through a very low-carbohydrate diet possibly reduces long-term glucose which in turn decreases insulin secretion. These lowered insulin levels may decrease androgen production and hyperandrogenism symptoms in women with PCOS [20].

Previous research on type 2 diabetes suggests that a very low-carbohydrate (VLC) diet with less than 20% of calories from carbohydrates would be even more effective for improving metabolic health in people with PCOS [22–26]. A VLC diet is designed to reduce glucose and insulin levels more significantly than other types of lower carbohydrate diets. For example, a meta-analysis of trials in adults with type 2 diabetes found that the greater the carbohydrate restriction, the greater the blood glucose control [27]. For example, VLC diets that omit dairy products have shown to reduce overall postprandial insulin production in comparison to VLC diets that include dairy [28]. Ultimately, research on low carbohydrate diets suggest that a non-dairy VLC diet is a promising for glucose control in people with PCOS because it is likely to generate less insulin that diets that do not restrict carbohydrates as much or at all.

Although reviews note that a VLC diet has shown promise for type 2 diabetes, prediabetes, obesity, and cardiovascular risk, evidence for PCOS is promising but still preliminary. The five published trials of a VLC diet in women with PCOS are small, with sample sizes of 5 to 40 participants [26, 28–32]. These VLC trials suggested improvements in glucose control and insulin resistance. We recently conducted a 4-month trial of 26 women with PCOS following a VLC diet and found that participants were highly satisfied with the intervention and experienced statistically significant improvements in outcomes including glycated hemoglobin (HbA1c; − 0.23%), body weight (− 7.1%), and quality of life [32]. The five previous trials and our pilot trial demonstrated that a VLC diet has potential for improving glycemic control for PCOS, suggesting that larger randomized trials should be conducted to confirm these results.

### Objectives {7}

To address the gaps in understanding of dietary interventions for PCOS management, we will be conducting a 12-month randomized comparative effectiveness trial for 184 people with a body weight in the overweight or obese category (BMI of 25–50 kg/m^2^ or 23–50 kg/m^2^ for Asians) with PCOS, comparing a VLC diet and a standard-of-care DASH diet. The DASH diet is recommended by experts for PCOS [17] and has been studied in two randomized trials in PCOS [33, 34]. The program for both groups is delivered online, as in our pilot study [32]. It includes coaching from trained study personnel, behavioral strategies to encourage dietary adherence such as mindfulness and relapse-prevention planning, and motivational and educational text messages to serve as reminders to participants.

This trial aims to compare the effects of a VLC diet and the DASH diet on clinical outcomes related to type 2 diabetes risk for individuals with PCOS, including glycemic control (HbA1c, our primary outcome), glycemic variability, conversion to normoglycemia, body weight, and body fat percentage. We also investigate whether either diet contributes to changes in symptoms of PCOS, such as testosterone levels, acne, hirsutism, and oligomenorrhea-anovulation. We hypothesize that the VLC version will lead to greater improvements in secondary health outcomes compared to the DASH diet. The trial also aims to explore satisfaction, acceptability, and feasibility of the dietary program through analysis of program satisfaction, dropout, adherence, side effects, quality of life, and barriers and facilitators to dietary adherence. We expect that both groups will be satisfied with the intervention and adherent to their assigned diet, in part due to the online approach with the supplementary adherence strategies, which has been tailored to better meet the needs of this population. We anticipate that the research will have an important impact on diet and lifestyle recommendations for this high-risk, understudied population.

### Trial design {8}

This trial is a single-site, parallel-group, randomized comparative effectiveness trial comparing two different dietary approaches for PCOS: a VLC diet vs the DASH diet. We will randomize a total of 184 adult women with PCOS using an allocation ratio of 1:1. This is a superiority trial. Assessments occur at baseline, 4 months, and 12 months after baseline.

## Methods: participants, interventions and outcomes

### Study setting {9}

The study clinical sites will include the main site at the University of Michigan, Ann Arbor, MI (L. Saslow, PI) and several remote clinical sites through the third-party organization Labcorp, which collects biological samples from participants who are not local to Ann Arbor, MI. The rest of the intervention will be administered remotely. We will recruit participants from the University of Michigan health system using outreach emails and letters to potentially eligible participants identified using electronic health records at these sites. We will also advertise on social media to reach potential participants outside of the University of Michigan health system. We aim to enroll a generally nationally representative sample of adult women with PCOS.

### Eligibility criteria {10}

Key inclusion criteria are defined based on whether the individual is taking hormonal birth control or birth control that alters menstrual cycle timing. For individuals who are not on hormonal birth control, inclusion criteria are oligomenorrhea-anovulation defined as spontaneous intermenstrual periods of < 21 days or > 35 days or a total of 8 or fewer menses per year and (2) hyperandrogenism, defined as total testosterone ≥ 35 ng/dL OR free testosterone > 4.0 pg/mL or a free androgen index > 1.5. For individuals who are taking hormonal birth control, key inclusion criteria include a history of oligomenorrhea-anovulation as described above prior to starting birth control; tests within the past 10 years from before the individual started birth control that show total testosterone, free testosterone, or free androgen index that meets the criterion 2 above; and tests within the past 10 years from before starting birth control that show other hormone levels as described in criterion 2 above.

For all individuals, other key inclusion criteria include BMI 25–50 kg/m^2^ or 23–50 kg/m^2^ for individuals who identify as Asian; 21–45 years of age; women or people assigned female at birth; access to the internet; ability to engage in light physical activity; willingness to be randomized to either dietary approach; and measured HbA1c at baseline of 5.3–9.0%.

Primary exclusion criteria include non-PCOS etiologies of anovulation and hyperandrogenism (such as Cushing’s disease, thyroid dysfunction, elevated prolactin levels, signs of a congenital adrenal hyperplasia, organic intracranial lesion like a pituitary tumor, or suspected adrenal or ovarian tumor secreting androgens); menopause or removal of both ovaries; history of type 1 diabetes; use of medications prescribed for weight loss or medications known to affect weight; current participation in another weight loss program or intervention; use of glucose-lowering medications other than metformin or medications known to affect metabolism, such as chronic oral corticosteroids; pregnant or planning to become pregnant during the intervention period; breastfeeding or less than 6 months postpartum; previous bariatric surgery or planning to have bariatric surgery during the study period; self-reported blood disorders that influence HbA1c, including frequent blood transfusions, phlebotomy, anemia, hemoglobinopathy, and polycythemia; residing outside the USA.

Additional exclusion criteria include the inability to read, write, or speak English; the inability to provide informed consent; adherence to a vegan or vegetarian diet; difficulty chewing or swallowing; lack of influence over what foods are purchased, prepared, and/or served or inability to follow dietary advice due to lack of money or other resources; above weight limit (500 lbs) in order to be appropriate for the dual X-ray absorptiometry scan; self-report of alcohol or substance use disorder within the past 5 years, including current at-risk drinking based on an Alcohol Use Disorders Identification Test (AUDIT) score of 15 or higher; Renal disease: BUN > 30 mg/dL or serum creatinine > 1.4 mg/dL in our screening blood tests or history of kidney stones; an untreated eating disorder or unstable serious mental illness (such as severe depression (score of 20 or greater on the Patient Health Questionnaire 8 (PHQ8)), bipolar or schizophrenic disorder with psychosis); use of warfarin; chronic kidney disease, stage 4 or higher; any other concerning values in baseline labs such as tests indicating triglycerides of 600 mg/dL or higher, baseline uncorrected thyroid disease, abnormal potassium levels, or baseline aspartate aminotransferase (AST) or alanine aminotransferase (ALT) > 2 times normal levels; or any condition for which the study team deems participation to be unsafe or inappropriate.

### Who will take informed consent? {26a}

A member of the study team who is approved by the institutional review board and trained in clinical trial ethics will conduct informed consents via Zoom video call. Prior to obtaining consent, the team member will lead the potential participant through a detailed review of the consent document via Zoom video in a private room. They will answer questions from participants and explain that participation in the study is voluntary.

### Additional consent provisions for collection and use of participant data and biological specimens {26b}

We will collect blood samples to assess outcomes such as HbA1c, high sensitivity C-reactive protein, lipids, and insulin resistance. However, the biological samples will not be stored after results are recorded.

## Interventions

### Explanation for the choice of comparators {6b}

The study will compare the effects of two diets on glucose regulation and PCOS symptoms: a very low-carbohydrate (VLC) diet and the Dietary Approaches to Stop Hypertension (DASH) diet. The diets vary in their approach to regulating insulin levels, glucose control, and weight loss, which are key targets for managing symptoms of PCOS [24–26]. Both diets have been recommended by groups of healthcare professionals as nutrition advice for women with PCOS, but experts disagree on which diet is best practice for nutrition advice to manage PCOS [17, 18, 20]. The study aims to address this knowledge gap by providing rigorous, long-term data comparing the diets’ effects on glucose control and PCOS symptoms.

### Intervention description {11a}

Participants will follow either the VLC or DASH diet for 12 months with guidance from a diet coach and 24 pre-recorded video sessions. They will be able to email their coaches at any time with questions, and they will have the option to attend monthly group video calls. Participants will also be able to request one-on-one calls with their coach at any time during the program. Regardless of how often participants reach out to their coaches, the coaches will reach out to each participant via email at least once biweekly during the program.

The participant will receive videos, informational handouts, and check-in surveys on a weekly basis for the first 4 months. Thereafter, they will receive videos and handouts monthly, and the check-in surveys will be distributed biweekly. The videos and handouts will teach about the assigned diet, skills to cope with stress and changing one’s diet, mindful eating and exercises to improve self-awareness, and information about PCOS and theory of the diet’s physiological influence on the condition. The surveys will ask questions about the participant’s dietary adherence, side effects and symptoms, program enjoyment, medication changes, and pregnancy status. To facilitate their transition to the new diet, participants will receive cookbooks throughout the study that correspond to their assigned diet. Participants will receive motivational texts and will be encouraged to self-monitor their eating patterns through food tracking and weighing themselves on a standard scale provided by the study throughout the intervention.

#### Very low-carbohydrate diet

The VLC diet aims to reduce carbohydrate intake to 20–35 g of non-fiber carbohydrates daily. Most calories are derived from meat, fish, eggs, fats, nuts, seeds, oils, leafy or other low-carbohydrate vegetables (such as spinach, lettuce, asparagus, eggplant, cabbage, kale, brussels sprouts, green peppers, and green beans), and low-carbohydrate fruits (such as raspberries and blackberries). Participants are advised to eliminate most starches and sweets such as potatoes, rice, pasta, bread, donuts, and sugar-sweetened beverages. Participants will be asked to avoid dairy. Participants will be advised to eat a moderate amount of protein with each meal and derive their remaining calories from fat.

The VLC diet is designed to lower carbohydrate intake to a point that induces a low level of ketone production. Nutritional ketosis may serve as a marker indicating that insulin levels are reduced enough to allow the body to begin using fat as a key source of energy, reducing inhibition of lipolysis by insulin. When this occurs, some fats are turned into ketones, which serve as a readily used fuel [18]. Participants randomized to this diet group are mailed urine ketone strips. They will be encouraged to use ketone urine test strips at the beginning of their time following this dietary approach to help them gauge whether they are achieving nutritional ketosis.

#### DASH diet

The DASH diet is recommended by some experts for management of weight and glucose control in women with PCOS [17]. It is a low-fat (20–30% of daily calories) and low-sodium (< 2300 mg daily) diet in which participants will be encouraged to eat fruits, vegetables, low-fat dairy foods, whole grains, lean meat and fish, and foods with little to no sugar, saturated fats, or oil. Participants will be taught about serving sizes and the appropriate number of servings to consume per day. The DASH diet is designed to lower the consumption of calories and saturated fat as the method for weight loss.

#### Physical activity goals

Participants will be encouraged to engage in physical activity that they enjoy for an average of 15–30 min per day. This recommendation is adapted from current guidelines for people with PCOS [35] and for diabetes prevention [36], which recommends 150 min of exercise per week.

#### Behavior goals

The primary behavior goal for this study is dietary adherence. The study is designed to encourage dietary adherence through support and education from coaches and through teaching ways of thinking that promote intentional eating habits. Mindfulness and positive affect will be taught through the sessions as optional behavior goals.

### Criteria for discontinuing or modifying allocated interventions {11b}

Serious adverse events resulting from the intervention are not expected. If a serious adverse event occurs, participants will stop the intervention immediately and will be included in the intention-to-treat analysis for the primary endpoint. Criteria for discontinuing or modifying the intervention include changes in the therapeutic plan of participants, such as new weight loss medications (other than metformin) or other medications that are not permitted; starting SGLT-2 inhibitors, as they increase the risk of ketoacidosis; the development of other concerning conditions such as severe depression or an untreated thyroid condition; or a positive pregnancy test. Participants are informed that they may refuse to answer any questions asked as part of the outcome measures by the study team and that they may withdraw their consent to participate in the study at any time.

### Strategies to improve adherence to interventions {11c}

Both dietary programs include strategies to improve adherence, including mindfulness, encouragement, and reminders, and tips for seeking support from loved ones. These are listed under psychological topics in Table 2.

### Relevant concomitant care permitted or prohibited during the trial {11d}

Participants will be instructed to work with their primary care providers about any existing issues, and primary care providers will be informed of this trial, as well. For all eligible participants, all blood test results will be sent to participants, and they will be advised to share blood test results with their primary care providers throughout the study.

Some side effects can be expected when changing one’s diet or losing a significant amount of weight. Participants may be directed to their primary care providers if symptoms are long lasting or are not able to be self-managed. Possible side effects include constipation, headaches, muscle cramps, bloating, dizziness, rash, hypoglycemia, hyperlipidemia, hypotension, kidney stones, and hair loss. All participants receive educational materials that will describe symptom self-management. They are asked to report symptoms in their check-in surveys to monitor for concerning side effects.

It is expected that some participants from either group may advance to type 2 diabetes while on this trial. If primary care providers decide to start their patient on glucose-lowering medications (except SGLT-2 inhibitors), those participants will be allowed to remain in the trial. Participants who start SGLT-2 inhibitors will be removed from the trial due to concerns for increased risk of ketoacidosis. Study physicians will use their judgement to determine if participants must be removed from the trial due to the development of other exclusionary diagnoses, etc.

Depression is common in women with PCOS. We assess depressive symptoms at baseline, month 4, and month 12. Participants with severe depression (a score of 20 or greater on the PHQ-8 during the screening survey) are ineligible and are referred to mental health resources. Participants with any PHQ-8 score between 10 and 20 are eligible but will also be referred to mental health resources. Participants with any PHQ-8 score below 10 are not referred to mental health resources, as the intervention itself contains positive affect skills which have been found to be effective for helping people with elevated depression, such as in a previous trial of the PIs [37]. If participants mention severe depression or suicidal ideation throughout the trial, a clinical psychologist will provide triage evaluation.

### Provisions for post-trial care {30}

We will not provide post-trial care. We do not anticipate harm and therefore no compensation for harm due to trial participation.

### Outcomes {12}

#### Primary outcome

##### HbA1c

According to standards for type 2 diabetes clinical care, HbA1c is the most widely accepted measure of overall glycemic control [38]. We aim to understand how these diets impact overall glycemic control via measurement of HbA1c, as people with PCOS are at greater risk for insulin resistance, poor glycemic control, and development of prediabetes or type 2 diabetes [9]. We measure HbA1c levels using standard immunoturbidimetric assay methods and quality control measures at a Clinical Laboratory Improvement Amendments (CLIA) certified lab (i.e., Labcorp). Our primary outcome is change in HbA1c from baseline to 12 months, and our secondary outcome is change in HbA1c from baseline to 4 months.

#### Secondary outcomes

##### Conversion to normoglycemia

Of critical concern is whether participants can convert to normoglycemia, having an HbA1c level of < 5.7%. In addition to measuring overall change in HbA1c, we record whether participants have converted to or maintained normoglycemia at the 4- and 12-month marks.

##### Glycemic variability

Greater glycemic variability may lead to complications [39], and intraday blood glucose variability is greater in people with prediabetes compared to people with normal glycemic levels [40]. We use continuous glucose monitors to compare measures of glycemic variability at baseline and 12 months. For in-person participants, we place a blinded Abbott Libre Pro continuous glucose monitoring device on a patient’s upper arm and will instruct participants to leave the monitor on for 14 days. Others are given the option to place a continuous glucose monitor at home with the assistance of a trained staff member on a video call.

##### Serum insulin and insulin resistance

Fasting insulin and glucose are used to calculate Homeostatic Model Assessment-Insulin Resistance (HOMA-IR). We measure fasting insulin and glucose at baseline, 4 months, and 12 months using standard assays from LabCorp to determine HOMA-IR, a widely used method of estimating insulin resistance from a single fasting blood draw [41].

##### High sensitivity to C-reactive protein

High sensitivity C-reactive protein (hsCRP) is an important acute inflammation protein and measure of inflammation which is associated with increased risk of cardiovascular disease [42]. Increased inflammation is associated with metabolic syndrome, and emerging evidence suggests that it may be a factor in the development of macrovascular disease in type 2 diabetes, which is more likely in patients with PCOS [43]. Beta hydroxybutyrate, the ketone body that is most abundant in the blood in patients following a ketogenic diet, has been shown to inhibit inflammation by disrupting the inflammatory response [44, 45]. A ketogenic diet is also associated with a reduction of reactive oxygen species, which are linked to inflammatory diseases [46]. We assess hsCRP using standardized methods in a CLIA certified laboratory (Labcorp).

##### Lipids

The ketogenic diet increases the proportion of calories derived from fat compared to a conventional diet such as the DASH diet. The greater intake of fat has prompted concerns about the effects of a ketogenic diet on blood lipids, especially LDL cholesterol. We aim to understand the possible impact of the ketogenic and DASH diets on macrovascular health in PCOS, and we include measurements of triglycerides and major plasma lipid fractions (HDL, LDL, triglycerides). We measure triglycerides and fractionated cholesterol using Labcorp’s Nuclear magnetic resonance (NMR) LipoProfile [47]. This advanced lipid assay provides measures that are more tightly tied to elevated cardiovascular risk than conventional lipid assays [48].

##### Body composition

Weight loss may involve the loss of lean body mass, fat body mass, or both, and each form of weight loss has a different association with health outcomes. In particular, a lower proportion of lean to total body mass is associated with greater mortality [49]. For participants local to Michigan, we measure body composition using Dual-Energy X-ray Absorptiometry (DEXA) scans.

##### PCOS symptoms

At baseline and at 4 and 12 months later, we will assess PCOS-related quality of life using the PCOSQ [50], acne using the Global Acne Severity Scale (Global Evaluation of Acne, or GEA scale) [51] hirsutism using a modified version of the Ferriman-Gallwey score [52], and cycle regularity through participant reports on cycle length, duration, and frequency.

#### Other assessments

##### Health-related quality of life

At baseline and at the 4- and 12-month checkpoints, we assess health-related quality of life using the PROMIS-29. This instrument is a collection of 4-item measures for broad domains of health-related quality of life including anxiety, depression, fatigue, pain interference, physical function, sleep disturbance, and ability to participate in social roles and activities, as well as a single pain intensity item [53].

##### Diet-related symptoms

We monitor for possible side effects throughout the trial. At baseline and at 4 and 12 months, we will also assess physical symptoms using a self-report scale.

##### Dietary adherence and changes

We assess dietary adherence with one unannounced 24-h dietary recall at baseline, 4, and 12 months. For each recall, we measure the number of calories consumed of each macronutrient and the percent that each contributes to total calories. For participants in the low-carbohydrate group, blood ketone levels are a biomarker to help assess whether target levels of carbohydrate restriction have been achieved. We assess fasting β-hydroxybutyrate at baseline, 4 months, and 12 months for all participants.

##### Session attendance and study dropout

We track attendance by monitoring whether participants have opened their session emails and whether they have completed their check-in surveys. Participants are considered dropouts if they ask to be withdrawn from the trial (or if they have been removed from the trial for any reason).

##### Program satisfaction

Using the Client Satisfaction Questionnaire [54], we assess intervention satisfaction at 4 and 12 months. It asks questions such as, “Which parts of the program have been most helpful to you?” and “On a scale from 0 to 10, how likely would you be to recommend our program to other people you know with PCOS?”.

##### Qualitative exploration of participants’ perspectives

We conduct optional qualitative semi-structured interviews with participants at 4 and 12 months after baseline. Interviews explore participant experiences, particularly their perceptions of the barriers to and facilitators of their ability to make long-lasting dietary changes.

##### Weight

Moderate weight loss is associated with improved glycemic control in type 2 diabetes [55]. Local participants are weighed at their in-person DEXA appointments at baseline and 12 months. At 0, 4, and 12 months, we collect measurements from their home scale that was provided by the study. The participants who do not come to the in-person appointments only have weight measurements from the home scale provided by the study.

#### Moderators

##### Body mass index

We will measure participants’ height and weight at baseline and 12 months and assess change in BMI.

### Participant timeline {13}

#### Screening and enrollment procedures

Enrollment occurs on a rolling basis. Participants who were ineligible for prior recruitments based on their blood work may be retested for eligibility later. Eligible participants start the program on the Sunday after they are randomized.

Participants are directed via recruitment materials and the study website to a screening survey through the online platform REDCap. The screening survey briefly describes the trial, asks for limited consent, and asks questions to obtain information regarding inclusion and exclusion criteria. The survey contains contact information for the study staff so that potential participants may contact the study team with any questions. The study staff follow up with potentially eligible participants via email, and they ask questions to verify eligibility. For any participant who scores greater than or equal to a ten on the PHQ-8, we will provide an automatic message directing them to mental health resources.

Participants who are determined to be potentially eligible based on the screening survey receive a study orientation video from the study team via email. The video gives a brief overview of the study interventions, procedures, and the expectations of participants. The video is followed by a short survey to assess comprehension, and a consent form to be reviewed and signed by the participant to move forward with a blood draw screening (described below).

After signing a consent for a baseline blood draw, participants receives an order for the following lab tests at their local Labcorp location: (1) total testosterone*; (2) free testosterone; (3) sex hormone-binding globulin; (4) comprehensive metabolic panel (Albumin, Albumin/Globulin Ratio (calculated), Alkaline Phosphatase, ALT, AST, BUN/Creatinine Ratio (calculated), Calcium, Carbon Dioxide, Chloride, Creatinine with GFR Estimated, Globulin (calculated), Glucose, Potassium, Sodium, Total Bilirubin, Total Protein, Urea Nitrogen); (5) thyroid-stimulating hormone (TSH); (6) HbA1c; (7) fasting insulin; (8) high-sensitivity C-reactive protein; (9) NMR LipoProfile with lipids; (10) beta hydroxybutyrate (ketone, important for VLC diet); (11) follicle-stimulating hormone (FSH); (12) estradiol; (13) luteinizing hormone (LH); (14) prolactin*; (15) dehydroepiandrosterone sulfate* (DHEAS); and (16) fasting 17-hydroxyprogesterone* (17-OHP). Tests marked with an asterisk will only be taken for individuals who are not on hormonal birth control because the hormone levels may be altered as a result of taking hormonal birth control. The participants who are taking hormonal birth control are required to have the necessary lab results for the tests marked above with an asterisk from within the past 10 years (when they were not using hormonal birth control).

If the participant’s blood draw data meet the inclusion criteria, the participant attends a virtual visit with a trained study team member to complete the full study informed consent. The study team member gives the participant a detailed review of the study, including procedures, expectations, risks, and benefits. The team member clearly explains that study participation is completely voluntary and that the participant may leave the study or refuse to participate at any time. Individuals who are still interested in the study upon reviewing the consent document with the study team member provide written informed consent using REDCap, and the team member helps the participant schedule their in-person appointment for follow-up testing. The study team member collects other information during the call, including availability for 24-h dietary recall phone calls. Participants may also consent to additional, optional research tasks during the visit, including optional, audio-recorded qualitative interviews at 4 and 12 months.

#### Baseline measurements and procedures

After completing the trial consent visit, participants are invited to complete baseline assessments before being randomization to one of the two diet groups.

Participants who live within driving distance of Ann Arbor attend an appointment at the Michigan Diabetes Research Center’s Clinical Research Unit, where the following assessments and procedures will be performed:

- Pregnancy test, which must be negative to proceed with enrollment
- DEXA scan
- Modified Ferriman-Gallwey score test for hirsutism
- The GEA scale
- Body weight and height
- Placement of a continuous glucose monitor (CGM), which is worn for 14 days and mailed back to the study team. The CGM does not give feedback to the participant, so they are blinded to the results. Per the instructions for the Libre Sensors being used, participants are instructed to not take more than 500 mg of vitamin C per day or more than 650 mg of aspirin per day while wearing the sensor during the study period.

Participants who are not in driving distance of Ann Arbor do not have an in-person appointment. They complete the following assessments and procedures at home:

- Body weight and self-reported height
- Optional at-home GEA scale exam using photos and video of the face
- Optional self-placement of a CGM with the assistance of a trained staff member on video call.

All participants receive an unannounced phone call from a study team member performing a 24-h dietary recall, in which the participant reports everything they ate the day prior. They also complete an online baseline survey that measures health-related quality of life, reward-based eating, and exercise habits.

The study team informs primary care providers and specialists (as needed) about the patient’s participation in this trial via a HIPAA-compliant fax. Primary care providers are asked to respond within 2 weeks of receiving the message if they believe that it is unsafe for the participant to move forward with the study. If the primary care provider does not respond or says that participation is acceptable, then the participant may move forward with the study. The primary care providers are notified that participants are expected to work with them to manage medications and preexisting issues, and they are informed that participants may come to them with side effects and blood test results from the study.

Participants are randomized to either diet upon completion of all assessments and activities listed above. Randomization is conducted using a computer-generated permuted block randomization process for well-balanced assignments and minimized ability to anticipate assignment. Although treatment condition is apparent to participants and researchers, outcome assessment and data analysis are masked. We stratify by baseline HbA1c: less than 6.5% or 6.5 and higher.

#### Interventions

The participants receive body weight scales in the mail. These scales automatically provide the study team with weights taken with no set up required by the participant. Once randomized, participants in the VLC group also receive ketone strips to test for urinary ketones at certain points in the study. The intervention begins the Sunday after a participant is randomized. They are sent the pre-recorded videos, reading materials, check-in survey, and home assignments via email on a weekly basis for the first 4 months, and monthly thereafter. The class content for each weekly and monthly session is detailed in Table 1. Participants have the opportunity to set up one-on-one calls with the diet coach and to attend group question and answer sessions with the coach. Coaches email participants at least biweekly.

**Table 1:** Session schedule for common elements in both intervention arms

Session	Dietary topics	Psychology topics
**Core sessions**		
#1	Study description, background on PCOS, rationale of assigned diet, basic information about assigned diet, changing snacks and breakfasts, calculating net carbs and calories, side effect guide	Connecting with community
#2	What happens to body when eating assigned diet, changing lunches, tracking diet, reading nutrition labels, menu makeover, tracking urinary ketones (for keto group)	Mindful eating
#3	Changing dinners, eating on a budget, food substitutions	Awareness of cravings and triggers
#4	Set yourself up for success, sugar and artificial sweeteners	Awareness of hunger and fullness
#5	Diet-friendly foods, meal prepping and batch cooking, how to plan and eat when dining out or traveling	Small and doable goals
#6	Physical activity and sleep, dealing with setbacks and challenges	Relaxation through breathwork
#7	Recap of diet-friendly foods, weight loss plateaus	Self-compassion
#8	Guide for problem solving, sticking to the diet	
#9	Dealing with triggers, speaking with loved ones about support, holiday foods	Boosting mood by paying attention to “feel good” moments
#10	Self-sabotage	Supporting oneself, recognizing personal strengths and preferences
#11	New recipes, quick meals	Accessible and enjoyable activities for self-care
#12	Recovering from setbacks, hidden carbohydrates (for keto group)	Positive reappraisal
#13	Effect of insulin on weight, tips to stay successful, food sensitivities	Reframing unhelpful thoughts
#14	Keto-friendly foods at restaurants, health problems related to sugar	“Best possible self” activity and identifying goals
#15	Revisiting traveling while on a diet, review of information about assigned diet	Gratitude practice
#16	Tools to help with sticking to and enjoying the program	Three funny things activity
**Maintenance sessions**		
#1	Reflection on how diet is going and favorite recipes	Using HALT: Hungry/Habit, Angry/Anxious, Lonely, or Tired/Thirsty
#2	Substitutes appropriate for the diet	Managing stress through the four A’s: accept, alter, adapt, avoid
#3	Grocery store walkthrough	Using guiding thoughts and images for motivation
#4	Reviewing diet-friendly foods and reflecting on favorite foods	Mini meditations for calming and mindfulness during eating
#5	Reviewing sweeteners, hunger and cravings	Managing setbacks
#6	Make-ahead recipes	Non-food rewards
#7	Diet-friendly celebratory foods, dealing with parties and social situations with food	Exploring one’s values
#8	Review of diet-friendly foods, review of finding and tracking nutrition for foods	Reflecting on most helpful strategies

At month 4, participants complete the following measures:

- One dietary recall over the phone
- An online survey (same as the baseline survey with the addition of program satisfaction questions)
- Body weight on a scale at their home
- A fasting blood draw as described in the baseline assessments

At month 12, participants complete the above measures in addition to:

- For participants that live within driving distance to Ann Arbor, an in-person visit with the same measures and procedures as the baseline in-person visit.
- For participants who do not live within driving distance of Ann Arbor, the same body weight and height measurements and optional CGM placement as the baseline procedures.

At months 4 and 12, participants are offered the opportunity to complete an optional interview to explore their experience with their assigned diet and the program. Interviews take place over Zoom and are audio-recorded and transcribed.

Upon completion of the 12-month tasks, the participant has completed the program. They are instructed to continue their management of their condition with their healthcare team.

**Fig. 1:**
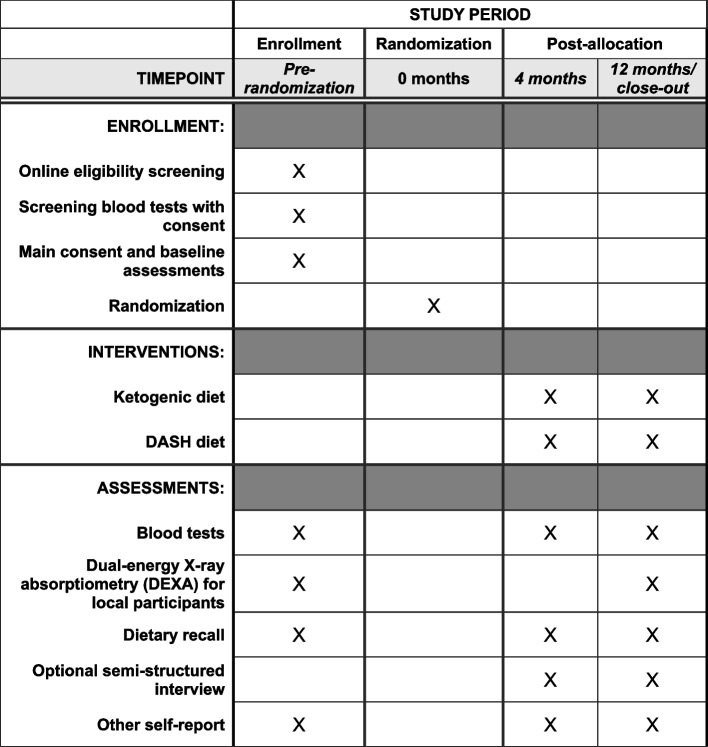
Schedule of enrollment, interventions, and assessments

Figure 1 shows the SPIRIT figure with the schedule of enrollment, interventions and assessments. Table 2 shows when participants complete these and other measures in the trial.

**Table 2:** Summary of measures by aim and month

Aim/domain	Measures	Baseline	4 mos	12 mos
**Aim 1: Health effects**				
*Physical health*	Blood tests for HbA1c (primary outcome), inflammation (hsCRP), comprehensive metabolic panel	x	x	x
	Glucose variability (via continuous glucose monitor)	x^a^		x^a^
*Body composition*	Dual-energy X-ray absorptiometry (DEXA)	x^a^		x^a^
*PCOS symptoms*	PCOS-related quality of life (PCOSQ)	x	x	x
	Acne, hirsutism	x^a^		x^a^
*PCOS-related hormones*	Total testosterone, free testosterone, sex hormone-binding globulin, thyroid-stimulating hormone, follicle-stimulating hormone, luteinizing hormone, estradiol	x	x	x
*Psychological and behavioral effects*	PHQ-8, health-related quality of life (PROMIS-29), and reward-based eating	x	x	x
**Aim 2: Potential adverse effects**				
*Lipids*	Nuclear Magnetic Resonance lipid profile (Labcorp LipoProfile)	x	x	x
*Diet-related symptoms*	Self-report	x	x	x
*Decreased vegetable consumption*	Fruit and vegetable consumption by 24-h dietary recall	x	x	x
**Aim 3: Assess possible moderators**				
*Hypothesized moderators*	Insulin resistance, body mass index	x	x	x
**Other outcomes**				
*Dietary adherence and changes*	Self-report and 24-h dietary recall	x	x	x
*Physical activity and sleep*	Self-report	x	x	x
*Intervention satisfaction*	Self-report, optional qualitative interviews (only once per participant)		x	x

^a^An asterisk indicates that this measure was optional or not possible for participants who were not able to attend an in-person appointment in Ann Arbor, MI

## Sample size {14}

According to a recent systematic review and network meta-analysis, which included 19 RCTs, a DASH diet was the most effective dietary pattern studied for reducing Homeostatic Model Assessment of insulin resistance [56]. Our pilot data and others suggest that in PCOS patients the VLC diet can reduce HbA1c by 0.3% and HOMA-IR [25, 32]. The difference in HbA1c between the VLC and high-carbohydrate studies is 0.2%. However, recognizing the small sample sizes for the pilot studies, we used 0.15% for our sample size calculation, as a conservative estimate of the observed effect.

To estimate the variance in the data, we noted that our pilot data and other studies of low-carbohydrate or VLC diets in individuals with PCOS had a standard deviation for the difference less than or equal to 0.3%. We also noted that the SD for the change in fasting glucose in studies of low-fat, higher-carbohydrate diets was comparable to the SD in VLC diets for individuals with PCOS [25, 28, 29, 33, 34, 57, 58]. Thus, we used 0.3% as the standard deviation in our sample size calculation. We used an alpha level of 0.05 and a power of 80%. To be conservative, we considered a *t*-test of the difference between the two groups at 12 months. Our proposed longitudinal model will use additional, repeated measurements and will increase the power of the test for a time-by-treatment effect [59]. The sample size needed to detect a 0.15% decrease in HbA1c with a standard deviation of 0.3% with an alpha of 0.05 and power of 80% using a two-sample *t*-test is 64 participants per group. Our 4-month pilot trial retained 81% of participants. Assuming a somewhat lower retention rate of 70%, the number needed to enroll is 92 per group or a total of 184.

## Recruitment {15}

We will recruit over 3 years using Michigan Medicine’s electronic medical records and social media advertisements on Meta and other platforms. We use electronic health records to identify potentially eligible participants, who are sent invitations to join the study either by email (e.g., MyChart emails) or through physical letters using the University of Michigan health system. We advertise on social media to reach community populations living outside of Michigan Medicine system areas within the USA.

## Assignment of interventions: allocation

### Sequence generation {16a}

Eligible participants are randomized in a 1:1 ratio to one of two groups based on a computer-generated permuted block randomization sequence for well-balanced assignments that minimizes the ability to anticipate assignment.

### Concealment mechanism {16b}

The allocation sequence is implemented via a software interface that conceals the sequence until the interventions are assigned to a participant.

### Implementation {16c}

The study statistician generated the allocation sequence. The allocation sequence is implemented via a database interface created for this study that conceals the sequence until an intervention arm is assigned to a participant. Study staff then inform participants of their intervention arm assignment.

## Assignment of interventions: masking

### Who will be unmasked {17a}

We do not mask participants to treatment arm before initiation of the intervention. We do not mask study staff to intervention group. Data analyses are performed by the study statistician, who is masked for planned primary and secondary outcome analyses, then may be unmasked. Laboratory staff collecting clinical outcomes (i.e., blood tests at Labcorp, DEXA scans, and 24-h dietary recall) are masked to group assignment. Participants also complete self-report questionnaires online, and the completion of these questionnaires are monitored by unmasked study staff.

### Procedure for unmasking if needed {17b}

We do not anticipate a need for unmasking the study statistician or abovementioned individuals, particularly as participants are aware of the nutritional intervention they are receiving, and we have thus not planned a procedure for unmasking.

## Data collection and management

### Plans for assessment and collection of outcomes {18a}

Study staff review all assessment data for accuracy and completion, and they monitor loss to follow-up and missing and incomplete data.

### Plans to promote participant retention and complete follow-up {18b}

Participants receive a BodyTrace Scale and cookbooks as part of the study. Participants receive a $20 Amazon gift card for completing baseline assessments, a $65 Amazon gift card for completing 4-month assessments and a $90 gift card for completing 12-month assessments. They will receive a $5 bonus incentive for attending optional Q&A sessions in months 5–12. The total bonus will be paid out once they complete their 12-month measures.

### Data management {19}

Trial data is collected by trained research assistants and study coordinators using questionnaires. Laboratory data (Labcorp) are transferred via electronic files and integrated into the study database, as are results from CGM’s and DEXA scans from University of Michigan Radiology. Protocol deviations are captured by regular review of cases during the enrollment process to ensure that eligibility criteria are met before randomization. Study data is stored using a HIPAA-compliant database (implemented in REDCap). The data system allows for specified ranges and automatic calculations to reduce entry errors. Data is cleaned by investigators upon completion of data collection to ensure high quality.

### Confidentiality {27}

All surveys and forms will be deidentified and coded with a unique participant number.

### Plans for collection, laboratory evaluation and storage of biological specimens for genetic or molecular analysis in this trial/future use {33}

Participants have blood samples drawn at baseline, 4 months, and 12 months. Blood samples are drawn, analyzed, and then destroyed by Labcorp.

## Statistical methods

### Statistical methods for primary and secondary outcomes {20a}

Outcome analyses will be performed by a dedicated biostatistician. An intention-to-treat analysis will be performed on all randomized participants and a per-protocol analysis will be performed on participants who adhere to their assigned diet. We define dietary adherence as reporting an average score of 5 or higher (scale of 1–7) for the level of dietary adherence on the final four surveys for which we asked about dietary adherence.

Descriptive statistics will be calculated for all outcome measures at each time point. Continuous variables will be reported using means, standard deviations, medians, and interquartile ranges, depending on the distribution. Categorical variables will be described using frequencies and percentages.

Our primary outcome is to compare HbA1c levels in participants in the two diet groups. Our primary analyses for HbA1c and other quantitative outcome measures will use random-intercept-random-slope mixed effects models to estimate differences between study arms in mean change in outcomes at 4 and 12 months. We will include several pre-specified covariates that we will adjust for to address any potential imbalances in randomization: sex, age, insulin use at baseline (yes/no), and education (as a marker of socio-economic status). When assessing outcomes such as HbA1c or weight over time, linear models that assume a constant association between time and outcome are unlikely to be appropriate. We anticipate much of the benefit to occur in the initial intervention period, with lesser benefit or even loss of benefit over time. To address this, we will use a linear spline of time, which uses knots to allow changes in slopes at key points, in this case, the end of the main intervention period. The overall issue of whether a very low-carbohydrate diet influences HbA1c primarily through weight loss or through other mechanisms is not the primary focus of this trial: both pathways are likely important, but the clinical result in glycemic control at the 12-month timepoint is the main focus. To explore the potential associations between weight loss and HbA1c; however, we will assess changes in HbA1c before and after statistically accounting for change in weight to help identify whether diet intervention group is affecting glycemic control via weight loss or via other mechanisms.

We will use similar analysis strategies for other continuous outcomes, using linear mixed-effects models with adjustments for the covariates noted for the primary outcome. We will use longitudinal models for continuous variables. For dichotomous variables (e.g., session participation), we will use generalized estimating equations to test the difference between treatment groups while accounting for the correlation over time. For categorical variables (e.g., side effects), we will use a chi-square test.

We will summarize symptom checklists by presenting the number and percent of participants who report each symptom more than 1 day in the previous month at baseline, 4 months, and 12 months. We will visualize data using stacked bar graphs that allow for visual comparison of presence and frequency of individual symptoms across intervention arms. We will use chi-squared tests to compare the percent of participants achieving diabetes remission or reversal.

We will use intent-to-treat and per-protocol analyses when analyzing satisfaction, acceptability, and feasibility of the program for women with PCOS.

### Sensitivity analyses

A set of sensitivity analyses will be conducted with models adjusting for baseline outcome (using an ANCOVA approach) and pre-specified baseline covariates as described above (age, insulin use at baseline, and education), to evaluate whether baseline differences impact estimates of treatment effects. Separate models will be run for each outcome time (4 months and 12 months), with baseline outcome measure included as a predictor, along with time, diet group, and their interaction, and covariates as described. A random intercept will be included to account for clustering by intervention group. Estimates of change within diet group, and differences between diet groups in change, will be derived from these models.

### Interim analyses {21b}

This trial has no planned interim analyses or stopping rules. Although our outcome, HbA1c, indicates long-term risk of clinical events due to diabetes, it is not an outcome that would justify early stopping rules such a trial. The Data and Safety Monitoring Board will review any intervention-related serious adverse events, to determine whether the study should be stopped.

### Methods for additional analyses (e.g., subgroup analyses) {20b}

We will explore whether baseline characteristics modify the benefits of diet group assignment on HbA1c by assessing whether there appear to be differences in the magnitude of HbA1c changes between diet intervention groups across three pre-defined sub-groups (levels of insulin resistance, levels of obesity, and women versus men). Linear mixed models similar to those describe above will be used, with the addition of candidate moderators (each in separate models), and interactions between the moderator, intervention arm, and time. These models will be used to estimate change in the outcome within subgroups and differences in change between subgroups. The focus of these exploratory analyses will be the magnitude and direction of change within and between subgroups. We will also report the statistical test on the interaction term, which represents an overall test of the moderation effect.

### Methods in analysis to handle protocol non-adherence and any statistical methods to handle missing data {20c}

We will examine patterns of missing data and proportions and compare baseline characteristics of participants with and without missing data to evaluate potential impact on estimates. In addition, our primary analyses for outcomes will use random-intercept-random-slope mixed effects models, to estimate differences between study arms in mean change in outcomes at 12 (primary) and 4 (secondary) months. Mixed effects models using maximum likelihood estimation are relatively robust to the effects of missing data, and they allow appropriate assessment of repeated measures.

### Plans to give access to the full protocol, participant-leveldata {31c}

Upon publication of the trial’s pre-specified outcomes, a de-identified dataset will be provided to other investigators upon reasonable request with the agreement of the trial steering committee, providing the request is in alignment with institutional review board protocols.

## Oversight and monitoring

### Composition of the coordinating center and trial steering committee {5d}

The primary decision-making body of this study is the investigative team comprising the principal investigator (PI; Saslow) and the co-investigators. The PI is responsible for the overall management of the study, coordinating the operation of the study, reviewing issues that arise in study conduct in between investigative team deliberations, and bringing relevant issues to the investigative team. The PI serves as the liaison with the funding body, including submission of annual reports and providing overall management of the fiscal and administrative operations, and is also responsible for the study coordination and implementation.

The trial steering committee consists of the PI, trial coordinators (AJ and SG), consulting MD (YS), and study statistician (DM). They meet weekly with study staff to discuss study implementation and adverse events. There is ongoing communication via email for needs such as enrollment questions and addressing issues such as suggesting medication changes for participants.

The project coordinators and research assistants are responsible for the day-to-day operations of the study, including screening and recruitment, data collection, dissemination of materials to participants, and intervention administration. They also coordinate institutional review board revisions, data monitoring reports, and document completion of necessary trainings for study staff. Staff are responsible for recruiting and screening the participants, obtaining informed consent with participants, and scheduling and conducting follow-up assessments and interviews. Interventionists (coaches) are responsible for the treatment implementation and for supporting participants, such as addressing questions and concerns. The project manager supervises the development of the study data tracking system and surveys.

### Composition of the data monitoring committee, its role and reporting structure {21a}

We created a 3-person Data Safety Monitoring Board that meets semiannually via videoconference. They review reports of recruitment, retention, and safety information.

### Adverse event reporting and harms {22}

In the case of a serious adverse event that is likely to be related to study participation, we will call for a special closed meeting of the Data and Safety Monitoring Board to review any needed changes or early stopping of the trial. Here, an adverse event includes any event that causes or increases the risk of harm to the participant or others. Serious adverse events include any events that result in death, inpatient hospitalization or prolongation of existing hospitalization, a persistent or significant disability or incapacity, or a congenital anomaly or birth defect. The study team reviews all potential adverse events reported by study participants and determines their relatedness to diet or study intervention, expectedness, and severity.

We will provide participants’ PCPs with information regarding the study once the participant has initiated the process for enrollment. If we recommend medication reductions, we will ask the participant to inform their PCP, and we also notify the PCP if the physician requests that we provide updates to them throughout their patient’s experience in the trial.

### Frequency and plans for auditing trial conduct {23}

Study investigators and the Data and Safety Monitoring Board will closely monitor the trial, meeting twice a year. The study team will provide annual progress reports to the institutional review board and to National Institute of Diabetes and Digestive and Kidney Diseases (NIDDK), the study sponsor.

### Plans for communicating important protocol amendments to relevant parties (e.g., trial participants, ethical committees) {25}

Any trial design changes, such as for trial eligibility, will be reviewed by the institutional review board. If the changes are approved by the institutional review board, they will be updated in ClinicalTrials.gov.

## Dissemination plans {31a}

The results of the trial will be presented at conferences, uploaded to ClinicalTrials.gov, and published in peer-reviewed publications. All final peer-reviewed manuscripts that arise from this proposal will be submitted to the digital archive PubMed Central. Wherever applicable, data will be deposited to appropriate public repositories.

## Discussion

The SUPER study is a 12-month randomized, comparative effectiveness trial comparing two dietary approaches to treating symptoms of PCOS, especially those that increase risk for type 2 diabetes, in adult women. The trial aims to provide a better understanding of the influence of diet, especially carbohydrate intake, on PCOS symptoms. Our protocol will compare the standard DASH diet and a very low-carbohydrate diet, which both show promise to improve glucose control and decrease the severity of PCOS symptoms. Glucose control will be measured primarily through change in HbA1c over the 12-month intervention, and PCOS symptoms will be measured via serum hormone levels and self-reported changes in menstruation, acne, and hirsutism. The study also aims to test the feasibility and acceptability of the dietary intervention for adult women with PCOS. We accompany the dietary approaches with informative sessions, coaching, and behavioral approaches to support dietary adherence. Feasibility, acceptability, and adherence will be measured via participant surveys of satisfaction and adherence. We hypothesize that the very low-carbohydrate diet will produce a greater improvement in glucose control, a decrease in severity of PCOS symptoms like acne, hirsutism, and oligomenorrhea-anovulation, and more weight loss.

One strength of the study is that we will recruit a nationwide sample, which allows our findings to be more generalizable to the population of people with PCOS within the USA. Limitations for this trial include the following: the inability to mask participants and administrative study staff to interventions; the age and BMI criteria limit the generalizability of our findings to only people aged 21–45 and with a body weight in the overweight to obese range; requirements of consistent internet access and technological proficiency; not providing people with meals increases the probability of lower adherence; English-only options for interventions. Exclusion criteria limit the generalizability of our findings, as we excluded individuals with self-reported food-insecure circumstances, pregnant or breastfeeding women, women who are trying to get pregnant, women who are taking hormonal birth control who do not have diagnostic testing from when they were naturally cycling, and people who take certain common medications to treat PCOS, like spironolactone and inositol. We aim to address the issue of masking as much as possible by masking the study statistician during analyses and masking study staff who perform assessments (dietary recalls, DEXA scans, blood draws). We aim to address additional biases as much as practically possible by randomizing and concealing of allocation where possible, employing strategies to minimize and manage incomplete outcome data, having appropriate duration of follow-up, and making a priori specifications of all primary and secondary outcomes as detailed in this study protocol and in our clinical trial registration.

PCOS is a common and burdensome disease that poses greater risk for fertility issues, type 2 diabetes, and other health problems [1–6]. Existing treatments are few and insufficient for treating the range of symptoms experiences by people with PCOS. Lifestyle interventions, such as dietary interventions, show promise in preventing type 2 diabetes and treat symptoms of PCOS. However, specific dietary recommendations vary among providers, and there is insufficient evidence to support VLC, DASH, or other diets as treatment for PCOS [2–5]. This trial addresses a knowledge gap in dietary influence on symptoms of PCOS and their risk for type 2 diabetes. To our knowledge, this is the first randomized, comparative effectiveness trial to compare the VLC and DASH dietary approaches in adult women with PCOS. We expect that the results of this trial will provide evidence of VLC and DASH diets’ influence on glucose control and PCOS symptoms in women with PCOS. We anticipate that the results may inform clinical practice guidelines for providers and patients with PCOS.

### Trial status

Protocol version 1.1; June 5, 2024. Recruitment was initiated on August 2022 and the approximate date for completion is December 2025.

## Data Availability

The final trial dataset will be available upon request.

## Abbreviations

BMI: Body mass index
CGM: Continuous glucose monitor
DASH: Dietary approaches to stop hypertension
DEXA: Dual-energy X-ray absorpiometry
HbA1c: Glycosylated hemoglobin
NIH: National Institutes of health
PCOS: Polycystic ovary syndrome
SUPER: Supporting understanding of PCOS education and research
VLC: Very low-carbohydrate
